# Coregulation of the cyclic lipopeptides orfamide and sessilin in the biocontrol strain *Pseudomonas* sp. CMR12a

**DOI:** 10.1002/mbo3.499

**Published:** 2017-06-15

**Authors:** Feyisara E. Olorunleke, Nam P. Kieu, Evelien De Waele, Marc Timmerman, Marc Ongena, Monica Höfte

**Affiliations:** ^1^ Laboratory of Phytopathology Faculty of Bioscience Engineering Ghent University Ghent Belgium; ^2^ Gembloux Agro‐Bio Tech University of Liège Gembloux Belgium

**Keywords:** cyclic lipopeptides, LuxR, *Pseudomonas*, transcriptional regulators

## Abstract

Cyclic lipopeptides (CLPs) are synthesized by nonribosomal peptide synthetases (NRPS), which are often flanked by LuxR‐type transcriptional regulators. *Pseudomonas* sp. CMR12a, an effective biocontrol strain, produces two different classes of CLPs namely sessilins and orfamides. The orfamide biosynthesis gene cluster is flanked up‐ and downstream by LuxR‐type regulatory genes designated *ofaR1* and *ofaR2*, respectively, whereas the sessilin biosynthesis gene cluster has one LuxR‐type regulatory gene which is situated upstream of the cluster and is designated *sesR*. Our study investigated the role of these three regulators in the biosynthesis of orfamides and sessilins. Phylogenetic analyses positioned OfaR1 and OfaR2 with LuxR regulatory proteins of similar orfamide‐producing *Pseudomonas* strains and the SesR with that of the tolaasin producer, *Pseudomonas tolaasii*. LC‐ESI‐MS analyses revealed that sessilins and orfamides are coproduced and that production starts in the late exponential phase. However, sessilins are secreted earlier and in large amounts, while orfamides are predominantly retained in the cell. Deletion mutants in *ofaR1* and *ofaR2* lost the capacity to produce both orfamides and sessilins, whereas the *sesR* mutant showed no clear phenotype. Additionally, RT‐PCR analysis showed that in the sessilin cluster, a mutation in either *ofaR1* or *ofaR2* led to weaker transcripts of the biosynthesis genes, *sesABC*, and putative transporter genes, *macA1B1*. In the orfamide cluster, mainly the biosynthesis genes *ofaBC* were affected, while the first biosynthesis gene *ofaA* and putative *macA2B2* transport genes were still transcribed. A mutation in either *ofaR1*,* ofaR2*, or *sesR* genes did not abolish the transcription of any of the other two.

## 1 INTRODUCTION

Cyclic lipopeptides (CLPs) are bacterial metabolites with biosurfactant activity composed of a cyclic oligopeptide lactone ring coupled to a fatty acid tail. The biosynthesis of CLPs is driven by nonribosomal peptide synthetases (NRPS), which are encoded by large gene clusters (Raaijmakers, de Bruijn, & de Kock, 2006). CLPs have drawn increasing interest for their versatile functions in plant beneficial *Pseudomonas*, which include involvement in biofilm formation, motility, and antimicrobial activity against a wide range of microorganisms including fungi, bacteria, viruses, and oomycetes (reviewed by Olorunleke, Kieu, & Höfte, 2015). Within the different CLP families, several CLP biosynthesis gene clusters have been fully characterized including orfamide, viscosin, massetolide, putisolvin, xantholysin, entolysin, and poaeamide, tolaasin, syringomycin, and syringopeptin, WLIP, arthrofactin, bananamide, thanapeptin, nunamycin, and nunapeptin (D'aes et al., 2014; De Bruijn et al., 2007; De Bruijn, de Kock, de Waard, van Beek, & Raaijmakers, 2008; Dubern, Coppoolse, Stiekema, & Bloemberg, 2008; Li et al., 2013; Vallet‐Gely et al., 2010; Zachow et al., 2015; Scherlach et al., 2013; Wang, Lu, Records, & Gross, 2006; Rokni‐Zadeh et al., 2012; Washio, Lim, Roongsawang, & Morikawa, 2010; Nguyen et al., 2016; Van Der Voort et al., 2015; Michelsen et al.*,* 2015).

The LuxR superfamily consists of transcriptional regulators that contain a DNA‐binding helix–turn–helix (HTH) motif in the C‐terminal region (Fuqua, Winans, & Greenberg, 1996). In this superfamily, four subfamilies can be distinguished based on domain architecture and the mechanism of regulatory activation. LuxR‐like proteins SalA, SyrF, and SyrG are a part of the fourth subfamily, which is characterized by the lack of any defined N‐terminal domain. These proteins have been associated with the regulation of the CLPs syringomycin and syringopeptin in *Pseudomonas syringae* pv. *syringae*, a plant pathogenic bacterium (Vaughn & Gross, 2016). In various other *Pseudomonas* species and strains, regulatory genes encoding similar LuxR‐like proteins are positioned up‐ and downstream of the CLP biosynthesis genes (De Bruijn & Raaijmakers, 2009a). Within several CLP families, the regulation of CLP biosynthesis has been attributed to LuxR‐type regulators including PsoR (putisolvin) in *P. putida* (Dubern et al., 2008), ViscA and ViscBC (viscosin) in *P. fluorescens* SBW25 (De Bruijn & Raaijmakers, 2009a), MassA and MassBC (massetolide) in *P. fluorescens* SS101 (De Bruijn & Raaijmakers, 2009b), ArfF (arthrofactin) in *P. fluorescens* MIS38 (Washio et al., 2010), EtlR (entolysin) in *P. entomophilia* L48T (Vallet‐Gely et al., 2010), WlpR (WLIP) in *P. putida* RW10S2 (Rokni‐Zadeh et al., 2012), XtlR (xantholysin) in *P. putida* BW11M1 (Li et al., 2013), and PcoR and RfiA (corpeptin) in *P. corrugata* CFBP 5454 (Strano et al., 2015).

In several *Pseudomonas* strains, the principal regulator of CLP biosynthesis is the GacA/GacS two‐component system since a mutation in one of both encoding genes leads to a loss in CLP production (De Bruijn & Raaijmakers, 2009a). The GacA/GacS system is known to activate small RNAs that bind to and sequester translational repressor proteins, which block the ribosomal binding sites in the mRNA of Gac‐regulated genes. Two small RNAs (sRNAs) and two repressor proteins, RsmA and RsmE, have been linked to the regulation of entolysin (Vallet‐Gely et al., 2010) and massetolide A biosynthesis (Song, Voort, et al., 2015). In the massetolide producer *P. fluorescens* SS101, these repressor proteins most likely block translation of the LuxR‐type transcriptional regulator, MassAR (Song, Voort, et al., 2015), by binding to a specific site called the GacA box. This site comprises a nontranslated leader sequence upstream of the AUG codon on the messenger RNA. In several CLP‐producing *Pseudomonas* strains, a GacA box is present upstream the LuxR regulators flanking the CLP biosynthesis gene cluster suggesting that other CLP‐producing *Pseudomonas* strains may show a similar regulation of lipopeptide biosynthesis (Song, Voort, et al., 2015).

Besides the GacA/GacS regulatory system, *N*‐acylhomoserine lactone (N‐AHL)‐mediated quorum sensing was shown to be required for viscosin and putisolvin biosynthesis (Cui, Harling, Mutch, & Darling, 2005; Dubern, Lugtenberg, & Bloemberg, 2006) in *P. fluorescens* strain 5064 and *P. putida* strain PCL1445, although this is not the case in certain other *Pseudomonas* strains (De Bruijn et al., 2008; Dumenyo, Mukherjee, Chun, & Chatterjee, 1998; Kinscherf & Willis, 1999). In *P. putida* strain PCL1445, two heat shock proteins DnaK and DnaJ located downstream of the Gac system were shown to regulate putisolvin biosynthesis (Dubern, Lagendijk, Lugtenberg, & Bloemberg, 2005). Recent studies on the genetic regulation of massetolide A biosynthesis in *P. fluorescens* SS101 revealed that the serine protease ClpP together with the chaperone ClpA regulates the biosynthesis of massetolides via a specific pathway involving the LuxR regulator (MassABC), the heat shock proteins DnaK and DnaJ, and proteins of the tricarboxylic acid (TCA) cycle (De Bruijn & Raaijmakers, 2009b; Song, Aundy, van de Mortel, & Raaijmakers, 2014; Song, Sundqvist, et al., 2015).

*Pseudomonas* sp. CMR12a is a biocontrol strain isolated from the cocoyam rhizosphere in Cameroon (Perneel et al., 2007). This strain produces two classes of CLPs namely orfamides and sessilins together with two types of phenazines, phenazine‐1‐carboxylate (PCA) and phenazine‐1‐carboxamide (PCN) (D'aes et al., 2014; Perneel et al., 2007). Orfamides are also produced by biocontrol agents belonging to the *P. protegens* group (Gross et al., 2007; Jang et al., 2013; Ma, Geudens, et al., 2016; Takeuchi, Noda, & Someya, 2014), while sessilins are structurally related to the tolaasins produced by the mushroom pathogen, *P. tolaasii*. Sessilins are important for biofilm formation, while orfamides are crucial for the swarming motility of CMR12a (D'aes et al., 2014) and both CLPs are important for biocontrol (D'aes et al., 2011; Hua & Höfte, 2015; Ma, Hua, Ongena, & Höfte, 2016; Olorunleke, Hua, Kieu, Ma, & Höfte, 2015).

**Figure 1:**
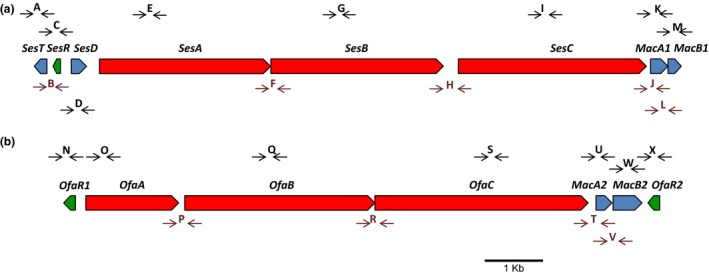
Schematic representation of sessilin (a) and orfamide (b) gene clusters of *Pseudomonas* sp. CMR12a. On both clusters, RT‐PCR amplicon positions are lettered A to X. SesT (NodT‐like outer membrane lipoprotein); SesR: LuxR‐type transcriptional regulator; SesD: SyrD‐like ABC transporter protein; OfaR1: LuxR‐type transcriptional regulator upstream of the orfamide gene cluster; OfaR2: LuxR‐type transcriptional regulator downstream of the orfamide gene cluster; MacA: periplasmic membrane protein; MacB: inner membrane protein. MacA1 and MacB1: associated with the sessilin gene cluster; MacA2 and MacB2: associated with the orfamide gene cluster. MacA1/MacA2 and MacB1/MacB2 share 78% and 80% identity, respectively

In CMR12a, sessilin biosynthesis is governed by three linked NRPS genes namely *sesA*,* sesB*, and *sesC* (Figure 1a) (D'aes et al., 2014). These genes are flanked upstream by a *nodT*‐like gene designated *sesT*, and downstream by *macA1* and *macB1* genes, which are probably involved in sessilin secretion. MacA and MacB are part of a tripartite secretion system involving an inner membrane protein (MacB), a periplasmic adaptor protein (MacA), and an outer membrane protein (NodT). Similar to sessilin, orfamide biosynthesis is governed by three linked NRPS genes namely *ofaA*,* ofaB*, and *ofaC* (Figure 1b) (D'aes et al., 2014). *MacA‐* and *macB‐like* genes putatively involved in orfamide secretion are located downstream of *ofaC*. Intriguingly, there is no *nodT*‐like gene in the orfamide gene cluster of *Pseudomonas* sp. CMR12a, while this gene is present in the orfamide gene clusters of *P. protegens* isolates (Ma, Geudens, et al., 2016). In addition, a LuxR‐type regulatory gene, *ofaR1*, is located upstream of the orfamide biosynthesis cluster and a second one, *ofaR2*, is situated downstream of the *macA2B2* genes, whereas a single LuxR‐type regulatory gene, *sesR*, is located upstream of the sessilin biosynthesis cluster next to the *sesT* gene (D'aes et al., 2014).

In this study, we hypothesized that in *Pseudomonas* sp. CMR12a, OfaR1 and OfaR2 regulate the biosynthesis of orfamides, whereas SesR is vital for sessilin biosynthesis. To test our hypothesis, site‐directed mutagenesis of the corresponding genes was conducted followed by biochemical and transcriptional analyses.

## 2 MATERIALS and METHODS

### 2.1 Bacterial strains and culture conditions

Bacterial strains, plasmids, and primers used in this study are listed in Table 1. *Pseudomonas* sp. CMR12a was cultured on Luria–Bertani (LB) agar plates or in liquid LB broth at 28°C. All molecular techniques were performed using standard protocols (Sambrook, Frithsch, & Maniatis, 1989). *Escherichia coli* strains were grown on LB agar plates or LB broth amended with appropriate antibiotics. *Saccharomyces cerevisiae* InvSc1 was cultivated on yeast extract–peptone–dextrose (YPD) (Shanks, Caiazza, Hinsa, Toutain, & O'Toole, 2006). *Escherichia coli* strain WM3064 was used as a host for the plasmids used in site‐directed mutagenesis.

**Table 1:** Strains, plasmids, and site‐directed mutagenesis primers used in this study^a^

Strains, plasmids, and primers	Relevant characteristics	Reference/Source
*Pseudomonas*		
CMR12a	PHZ^+^, CLP1^+^, CLP2^+^, wild type (Cameroon)	Perneel et al. (2007)
CMR12a‐ΔsesR	Mutant with deletion of *luxR* gene in sessilin cluster	This study
CMR12a‐ΔofaR1	Mutant with deletion of *luxR* gene, upstream orfamide cluster	This study
CMR12a‐ΔofaR2	Mutant with deletion of *luxR* gene, downstream orfamide cluster	This study
*P. protegens* Pf‐5	Orfamide, wild type	Loper and Gross (2007)
*Escherichia coli*		
DH5α	Host for cloning	Hanahan (1983)
WM3064	Strain for conjugation; λ pir, DAP auxotroph	Saltikov and Newman (2003)
*Saccharomyces cerevisiae* InvSc1	Yeast strain for *in vivo* recombination (*ura3‐52/ura3‐52* mutation)	Invitrogen
Plasmids		
pMQ30	Gene replacement vector for *Pseudomonas* species; *sacB*, URA3, Gm^R^	Shanks et al. (2006)
pMQ30‐ΔsesR	Vector for site specific mutagenesis of *luxR* gene in sessilin cluster	This study
pMQ30‐ΔofaR1	Vector for site specific mutagenesis of *luxR* gene, upstream orfamide cluster	This study
pMQ30‐ΔofaR2	Vector for site specific mutagenesis of *luxR* gene, downstream orfamide cluster	This study
pME6032	Expression vector with *tac* promoter; Tc^R^	Heeb, Blumer, and Haas (2002)
pME6032‐SesR	Expression vector with *sesR* of CMR12a under *tac* promoter; Tc^R^	This study
pME6032‐OfaR1	Expression vector with *ofaR1* of CMR12a under *tac* promoter; Tc^R^	This study
pME6032‐OfaR2	Expression vector with o*faR2* of CMR12a under *tac* promoter; Tc^R^	This study
Primers (5′ → 3′)		
OfaR1‐Up‐F	GGAATTGTGAGCGGATAACAATTTCACACAGGAAACAGCTGGAAGTCGTGAAAGGCCAGT	This study
OfaR1‐Up‐R	GCTGTTCTTGACGCTCAGGGAGGTTGCTGCTCAGACTCA (911 bp)	This study
OfaR1‐Down‐F	TGAGTCTGAGCAGCAACCTCCCTGAGCGTCAAGAACAGC	This study
OfaR1‐Down‐R	CCAGGCAAATTCTGTTTTATCAGACCGCTTCTGCGTTCTGATTTCAGTGTGCGACTCAATCC (885 bp)	This study
OfaR2‐Up‐F	GGAATTGTGAGCGGATAACAATTTCACACAGGAAACAGCTGGCTGCCTTCACCTTCTATGC	This study
OfaR2‐Up‐R	CTCACTCAGGTTTGCTGCTGATGACCTTGCCAATGTGAGG (883 bp)	This study
OfaR2‐Down‐F	CCTCACATTGGCAAGGTCATCAGCAGCAAACCTGAGTGAG	This study
OfaR2‐Down‐R	CCAGGCAAATTCTGTTTTATCAGACCGCTTCTGCGTTCTGATCGTCAGCCACCTGTACTTCA (896 bp)	This study
SesR‐Up‐F	GGAATTGTGAGCGGATAACAATTTCACACAGGAAACAGCTGCTTGAGGCCAAAGACCAGAC	This study
SesR‐Up‐R	CACTTGGTCAATCCATGTCG TGAATGCTGCTCGTCATTTC (953 bp)	This study
SesR‐Down‐F	GAAATGACGAGCAGCATTCACGACATGGATTGACCAAGTG	This study
SesR‐Down‐R	CCAGGCAAATTCTGTTTTATCAGACCGCTTCTGCGTTCTGATAACCAGCAACGTCAGGCTAT (863 bp)	This study

a PHZ, phenazines; CLP1, sessilins; CLP2, orfamides; Gm^R^, Tc^R^, Amp^R^, Km^R^, resistant to gentamycin, tetracyclin, ampicillin, kanamycin, respectively.

### 2.2 Analysis of CLP production

For LC‐ESI‐MS analyses, bacterial strains were grown at 28°C in six‐well plates with 2.5 ml LB broth per well. Cultures were maintained for variable time periods after which 1 ml of each was centrifuged at 18,900*g* for 4 min. Filter‐sterilized supernatants were subjected to reverse‐phase LC‐ESI‐MS as described by D'aes et al. (2014). Cells obtained after the centrifugation step were washed once with sterile distilled water resuspended in 1 ml of acetonitrile solution (50%) after which sonication was carried out for 30 s. Following centrifugation, the cell supernatant was filter sterilized and subjected to LC‐ESI‐MS analysis. Data generated from supernatant and cell analyses were processed to either extract chromatograms or obtain the relative production of sessilins and orfamides using the MassLynx V4.1 software.

### 2.3 Site‐directed mutagenesis

Site‐directed mutagenesis of the *ofaR1*,* ofaR2*, and *sesR* genes was performed based on methods described previously (D'aes et al., 2014). To construct each mutant, a fragment of the corresponding LuxR biosynthesis gene was deleted by allelic replacement with vector pMQ30 (Shanks et al., 2006). Primers used for polymerase chain reaction (PCR) and plasmids are described in Table 1. To obtain a deletion plasmid, two coding regions of each LuxR gene were amplified by PCR and these products were cloned next to each other by homologous recombination in *S. cerevisiae* InvSc1. This plasmid was mobilized into CMR12a by conjugation with *E. coli* WM3064 and selection on gentamycin. Subsequently, transconjugants that had lost the plasmid during the second crossover event were selected on LB with 10% sucrose after which gene deletion was confirmed by PCR and sequencing (LGC Genomics, Germany).

### 2.4 Construction of pME6032‐based vectors for complementation

A fragment containing the *luxR* gene was obtained by PCR with specific primers (Table 1). These PCR products were subsequently cloned in the expression vector pME6032 comprising the pTac promoter. The plasmids obtained, pME6032‐OfaR1, pME6032‐OfaR2, and pME6032‐SesR were transformed into *E. coli* WM3064 via heat shock after which transformed colonies were selected on LB agar plates supplemented with tetracycline 50 μg/ml. Correct integration of fragments was verified by PCR analysis, restriction analysis of isolated plasmids, and sequencing. These three pME6032‐based *E. coli* WM3064 vectors were transformed into the corresponding *Pseudomonas* sp. CMR12a LuxR mutants by conjugation. Transformed cells were selected on LB supplemented with 100 μg/ml tetracycline and the presence of pME6032‐OfaR1, pME6032‐OfaR2, or pME6032‐SesR was confirmed by PCR analysis using primers specific for pME6032 and the corresponding *luxR* gene.

### 2.5 White line‐in‐agar and swarming motility assays

The white line‐in‐agar test (Rokni‐Zadeh, Li, Yilma, Sanchez‐Rodriguez, & De Mot, 2013) was performed in triplicate on Kings’ B (KB) medium. Bacterial strains were cultured in LB broth for 16 hr and washed twice with saline solution (0.85%). The line of bacterial indicator strain (*P. protegens* Pf‐5) in the middle of the plates was made from three drops (5 μl per drop) of the suspension. Subsequently, 5 μl suspension of each test bacterial strain was spotted at both sides of the line within a 1‐cm distance. White precipitate formation in the agar was evaluated after 3 days of growth at 28°C.

For swarming motility assays, 5 μl suspension of each test strain was spotted in the center of LB plates comprising 0.6% agar, left to dry briefly and incubated at 28°C for 24 hr (D'aes et al., 2014). At least two replicates per strain were included, and experiments were repeated at least twice.

### 2.6 RNA extraction and reverse transcription‐PCR (RT‐PCR)

Bacterial cells were grown in still cultures using a six‐well plate containing 2.5 ml LB broth per well at 28°C. At 24 hr, growth of strains was determined by measuring optical density OD_620_ of 100 μl in a 96‐well plate using a Bio‐Rad 680 microplate reader after which 1 ml of cell culture was collected and spun down. Cells were frozen in liquid N_2_ and stored at −80°C. For the RNA extraction and complementary DNA (cDNA) synthesis, two biological replicates were used. RNA was isolated from frozen bacterial cells with the Trizol reagent (Sigma), followed by genomic DNA removal using the Turbo DNA‐free kit (Ambion/Applied Biosystems). cDNA was synthesized by using the GoScript Reverse Transcription System (Promega). cDNA with RNA equivalent of 100–200 ng was subjected to PCR with specific primers listed in Table S1. The thermal profile used consisted of an initial denaturation step at 95°C for 2 min, followed by 30 cycles of 94°C for 30 s, 54°C for 30 s, and 72°C for 1 min. The primer pairs were used to amplify cDNA obtained from transcripts corresponding to genes of the sessilin and orfamide biosynthesis gene clusters and their flanking genes including the *sesT*,* sesR*,* ofaR1*,* ofaR2*,* ofaABC*,* sesABC*, and the *macAB* genes. Transcripts covering adjacent gene pairs of the aforementioned genes were also amplified.

### 2.7 Bioinformatic analyses

LuxR‐like protein sequences for *Pseudomonas* sp. CMR12a were obtained from the nucleotide sequences of the sessilin and orfamide biosynthesis gene clusters with GenBank accession numbers JQ309920 and JQ309921, respectively. Other amino acid sequences used for phylogenetic analyses were collected from the National Centre for Biotechnology Information (NCBI) website. Characteristics of strains and protein sequences used in the phylogenetic analyses of LuxR proteins are presented in Table S2. Sequence alignments were made using Muscle (Edgar, 2004) via the software package MEGA6 (Tamura, Stecher, Peterson, Filipski, & Kumar, 2013). Phylogenetic tree was inferred by maximum likelihood (ML) using 1000 bootstrap replicates and was rooted with the LuxR (quorum sensing protein) from *Vibrio fischeri*. Proteins of *N*‐acyl‐l‐homoserine lactones (acyl‐HSLs)‐binding regulators of CMR12a, CmrR and PhzR (De Maeyer, D'aes, Hua, Nam, & Höfte, 2013), were included in this analysis.

Furthermore, bioinformatic tools were employed to check for the presence of Rsm binding sites upstream of the *ofaR1*,* ofaR2*, and *sesR* genes. The query search was conducted using the conserved motif 5′‐^A^/U CANGGANG^U^/A‐3′, where N denotes any nucleotide (Song, Voort, et al., 2015). Subsequently, similar nontranslated leader sequences flanking the LuxR transcriptional regulators of several CLP‐producing *Pseudomonas* strains were aligned with the three LuxR regulators of *Pseudomonas* sp. CMR12a.

## 3 RESULTS

### 3.1 Growth and production of sessilins and orfamides by CMR12a in shaken and still LB broth cultures

**Figure 2:**
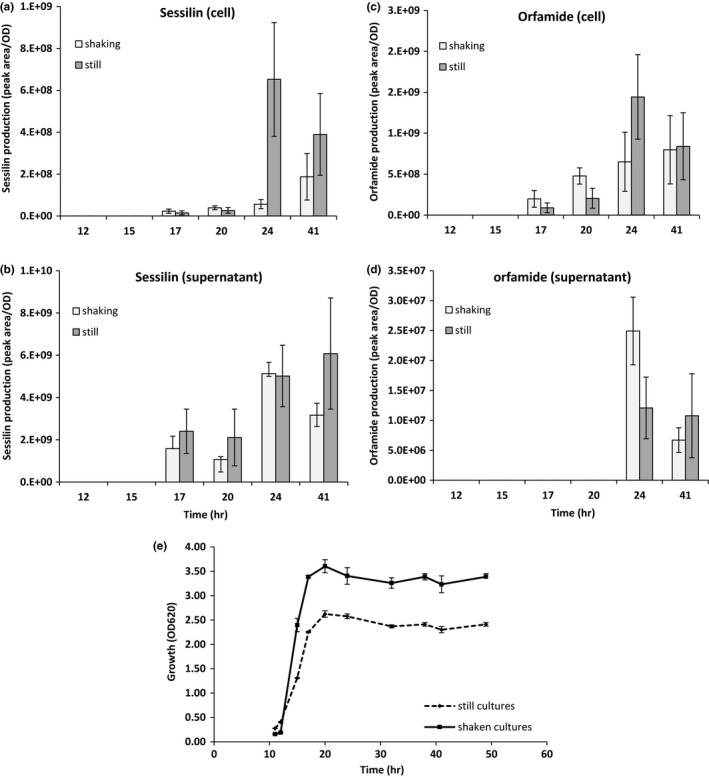
Quantification of sessilins and orfamides produced and secreted by wild‐type strain, *Pseudomonas* sp. CMR12a, in still and shaken growth conditions. (a) Sessilins in cells, (b) sessilins in supernatants, (c) orfamides in cells, (d) orfamides in supernatants, (e) growth curve of *Pseudomonas* sp. CMR12a over time points. Bacteria cultures were grown in still and shaking (150 rpm) LB broth conditions at 28°C. At each time point, cell density was measured spectrophotometrically (OD620) and mean values from three replicates were recorded. Time points were representative of different growth phases of *Pseudomonas* sp. CMR12a. 17 h: late exponential growth phase; 20 h: early stationary growth phase; 24 h: stationary growth phase; 41 h: death phase. For all graphs, different scales were used to represent peak area/OD. Values are means ± standard error (*n* = 3)

To quantify the production of sessilins and orfamides by CMR12a in shaking (150 rpm) and still LB broth culture conditions, filter‐sterilized supernatants and cells were collected at various time points, prepared and subjected to LC‐ESI‐MS analysis. Time points chosen—17, 20, 24, and 41 hr—corresponded to the late exponential growth phase, early stationary growth phase, stationary growth phase, and death phase of CMR12a. Analyses of relative CLP production (relative peak area/OD_620_) showed that in both culture conditions, coproduction of sessilins and orfamides started at 17 hr (Figure 2). Most of the sessilins produced were immediately secreted into the supernatant, while lower amounts were kept inside (Figure 2a and b). In contrast, orfamides were mainly retained in the cells (Figure 2c). Unlike sessilin secretion, the secretion of orfamides into the LB broth occurred 7 hr after the start of CLP production in both culture conditions (Figure 2d). Aerated cultures reached a higher biomass than still cultures (Figure 2e). In general, aeration had no strong effect on CLP production, although at 24 hr it seemed that more CLPs were retained inside the cell in still conditions.

### 3.2 Functional analysis of *luxR*‐type regulatory genes in sessilins and orfamides biosynthesis

**Figure 3:**
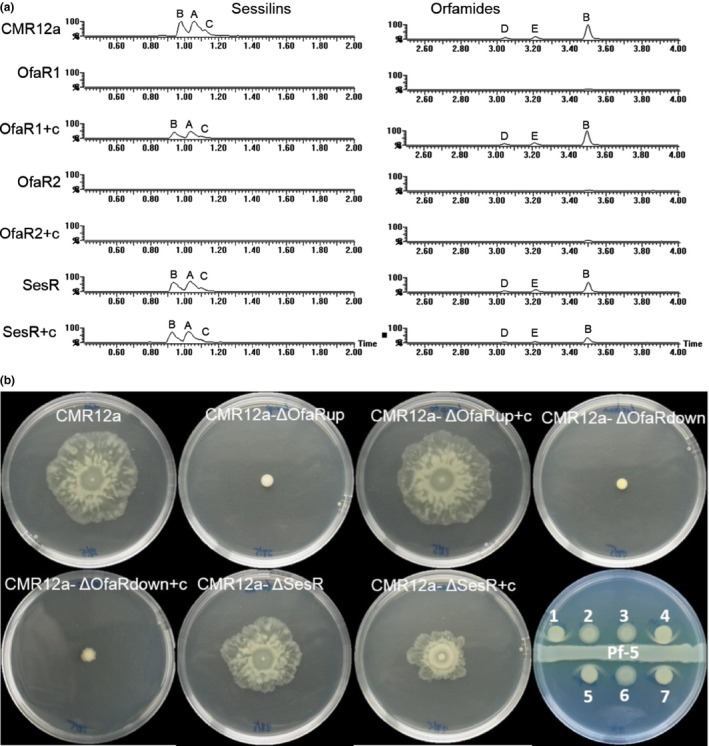
CLP characterization of CMR12a, LuxR mutants and complemented strains. (a) LC‐ESI‐MS chromatograms of cell‐free culture supernatants of wild‐type *Pseudomonas* sp. CMR12a, LuxR mutants, and complemented strains after 24 hr of incubation. Wild‐type produces sessilins (analogs—A, B, and C) and orfamides (analogs—B, D, and E). CMR12a: wild‐type *Pseudomonas* sp. CMR12a; OfaR1: OfaR1 biosynthesis mutant; OfaR1+c: complement of OfaR1 biosynthesis mutant; OfaR2: OfaR2 biosynthesis mutant; OfaR2+c: complement of OfaR2 biosynthesis mutant; SesR: SesR biosynthesis mutant; SesR+c: complement of SesR biosynthesis mutant. (b) Swarming ability of CMR12a and LuxR mutants on 0.6% LB agar and white line‐in‐agar tests on KB agar medium. Bacterial cultures were grown for 17 hr in LB broth and washed twice with saline solution (0.85%). Five microliter of the suspensions was spotted in the center of the plates and incubated at 28°C for 24 hr. For the white line test, the picture was taken 3 days after incubation at 28°C. (1) CMR12a, wild‐type *Pseudomonas sp*. CMR12a; (2) CMR12a‐ΔOfaR1, OfaR1 biosynthesis mutant; (3) CMR12a‐ΔOfaR2, OfaR2 biosynthesis mutant; (4) CMR12a‐ΔSesR, SesR biosynthesis mutant; (5) CMR12a‐ΔOfaR1+c, *ofaR1* gene complement of OfaR1 biosynthesis mutant; (6) CMR12a‐ΔOfaR2+c, *ofaR2* gene complement of OfaR2 biosynthesis mutant; (7) CMR12a‐ΔSesR+c, *sesR* gene complement of SesR biosynthesis mutant

LC‐ESI‐MS analysis revealed the complete abolishment of orfamide and sessilin production in the *ofaR1* and *ofaR2* mutants (Figure 3a). However, the mutant in the *sesR* gene, located upstream of the sessilin biosynthesis cluster, still produced sessilins and orfamides.

Additionally, quantitative measurements (relative peak area/OD_620_) of the two CLPs did not reveal any difference between CMR12a and CMR12a‐∆*sesR* (data not shown). Restored sessilin and orfamide production was observed in the complemented *ofaR1* mutant, but not in the complemented *ofaR2* mutant (Figure 3a).

Previous results showed that orfamides are important in the swarming motility of CMR12a and that sessilins and orfamides interact to give a white line on KB medium (D'aes et al., 2014). In order to ascertain the cessation of sessilin and orfamide production by the LuxR mutants of CMR12a, swarming motility and white line tests were conducted. Similar to CMR12a, the *sesR* mutant swarmed on 0.6% LB agar. However, *ofaR1* and *ofaR2* mutants did not exhibit swarming motility (Figure 3b). Complementation of the mutants with each of the corresponding target genes cloned into the stable vector pME6032 restored swarming motility in the *ofaR1* mutant, but not in the *ofaR2* mutant. The white line‐in‐agar formation is typical for CMR12a when it interacts with an orfamide producer such as *P. protegens* Pf‐5 and is indicative for sessilin production. In our study, CMR12a‐∆*ofaR1*, CMR12a‐∆*ofaR2*, and the complemented *ofaR2* mutants no longer secrete sessilins, since they did not give the white line‐in‐agar interaction when challenged with the orfamide producer, *P. protegens* Pf‐5. The white line‐in‐agar phenotype was observed, however, for CMR12a, CMR12a‐∆*sesR*, and the complemented *ofaR1* mutant strains (Figure 3b).

### 3.3 Transcriptional analysis of flanking and CLP biosynthesis genes in CMR12a and LuxR mutants

**Figure 4:**
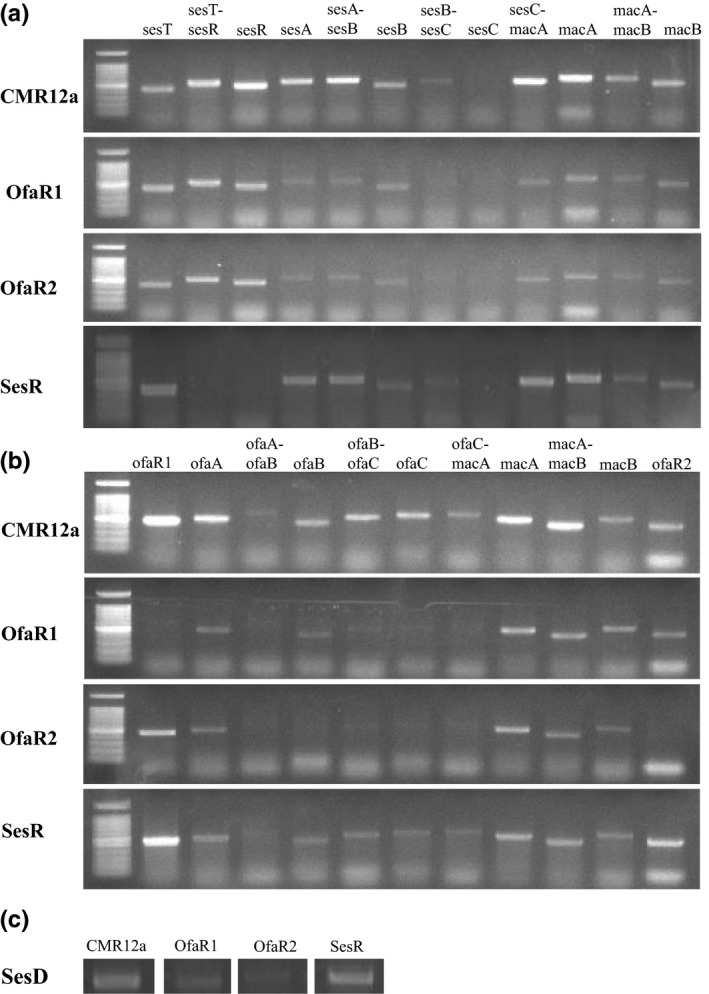
RT‐PCR analyses for the sessilin (a) and orfamide (b) biosynthesis gene clusters and flanking genes in CMR12a and LuxR mutants, (c) *sesD* (*syrD*‐like) gene associated with the sessilins gene cluster. Bacterial cells analyzed were collected from 24 hr culture of *Pseudomonas* sp. CMR12a and its LuxR mutants. For each gene within the sessilin and orfamide gene clusters, the same bacterial culture was analyzed in duplicate and representative results are shown for one experiment. Agarose gel results are shown for analysis of single genes together with gene coexpression to distinguish monocistronic and polycistronic transcription. Primers used are listed in Table S1 and the amplicon positions are as indicated in Figures 1a and b

Figure 1a and b show primer positions for RT‐PCR on the sessilin and orfamide gene clusters, respectively. In order to investigate the transcriptional analysis for *ofaABC*,* sesABC*, and their flanking genes, RT‐PCR was conducted for CMR12a and LuxR mutants using bacterial cell cultures which were grown in still LB cultures for 24 hr in two replicates (Figure 4a–c).

For the sessilin biosynthetic gene cluster, RT‐PCR analysis of the WT strain revealed the transcription of *sesA*,* sesB*, and flanking genes, *sesT*,* sesR*,* macA1*, and *macB1*, whereas *sesC* was not transcribed. Additionally, the coexpression of *sesT‐sesR*,* sesA‐sesB*,* sesB‐sesC*,* sesC‐macA1*, and *macA1‐macB1* gene combinations indicate that the *sesT*‐*sesR* genes on one hand and the *sesABC* together with *macA1B1* genes on the other hand are transcribed from a polycistronic operon (Figure 4a). In contrast, analysis of the CMR12a‐∆*ofaR1* mutant mainly revealed the transcription of *sesT‐sesR* and *macA1B1* genes. Furthermore, this mutant was characterized by the presence of weak *sesAB* transcripts. For the CMR12a‐∆*ofaR2* mutant, similar transcription results as the CMR12a‐∆*ofaR1* mutant were obtained. Additionally, RT‐PCR analyses of the CMR12a‐∆*sesR* mutant revealed similar results as the WT except for the expected absence of *sesT‐sesR* and *sesR* expression.

Transcriptional analyses of the orfamide biosynthesis gene cluster were also conducted after growing bacterial cultures for 24 hr. In the WT strain, *ofaR1*,* ofaA*,* ofaB*,* ofaC*,* ofaR2*, and the gene combinations of *ofaB‐ofaC* were clearly transcribed, whereas *ofaA‐ofaB* gave a weak transcript (Figure 4b). Additionally, the transcription of *macA2* and *macB2* and gene combinations of *ofaC‐macA2* and *macA2‐macB2* show that *ofaABC* and *macA2B2* are also transcribed from a polycistronic operon. For the *sesR* mutant, expression and coexpression analyses of all genes and gene combinations showed similar results with CMR12a. In contrast, the CMR12a‐∆*ofaR*1 mutant showed the transcription of *ofaA*,* macA2B2* and *ofaR2* coupled with weak *ofaB*, and *ofaB–ofaC* transcripts. More so, CMR12a‐∆*ofaR2* mutant only showed *ofaR*1, *ofaA*,* macA2B2*, and weak *ofaBC* transcripts (Figure 4b).

Furthermore, a mutation in either of the three LuxR‐type genes of CMR12a did not appear to abolish the transcription of the other (Figure 4A and B). OfaR1 and *ofaR2* mutants appeared to show a weaker transcription of the *sesD* (*syrD*‐like) gene, whereas the *sesR* mutant showed similar results with CMR12a (Figure 4c).

### 3.4 Phylogenetic analyses of LuxR‐type regulatory proteins associated with CLP gene clusters

**Figure 5:**
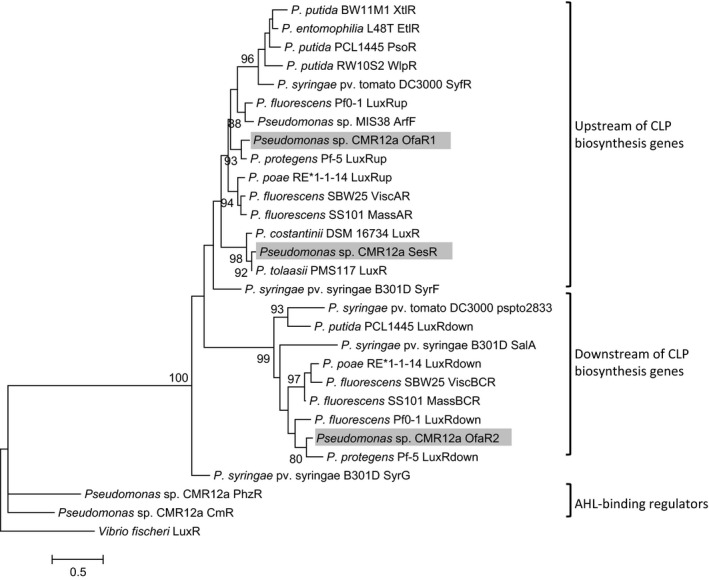
Phylogenetic analysis of the LuxR‐type regulators flanking the orfamide and sessilin biosynthesis genes of *Pseudomonas* sp. CMR12a (highlighted in gray). Also included in this analysis are the LuxR‐type regulators of other *Pseudomonas* CLP biosynthesis genes, and AHL‐binding regulators LuxR from *Vibrio fischeri,* and PhzR and CmrR from *Pseudomonas* sp. CMR12a. The dendrogram was generated by maximum likelihood using 1,000 resampled datasets. Percentage bootstrap values are indicated at branching nodes while the bar indicates sequence divergence

Phylogenetic analyses of the CLP cluster‐associated LuxR‐type proteins of CMR12a together with that of other *Pseudomonas* strains, showed several distinct clusters (Figure 5) as follows: OfaR1 and SesR proteins clustered together with other LuxR‐type regulators located upstream of CLP biosynthesis genes. Similarly, OfaR2 clustered with LuxR‐type regulators located downstream of the CLP biosynthesis genes. Specifically, SesR clustered with other LuxR‐type regulators within the tolaasin group, while OfaR1 and OfaR2 clustered with regulators which flank orfamide‐coding genes in other *Pseudomonas* strains including *P. protegens* Pf‐5 (Loper & Gross, 2007). The AHL‐binding regulators of CMR12a, CmrR and PhzR, formed a separate cluster together with the LuxR of *V. fischeri* indicating that they belong to a separate subfamily of regulators (Figure 5).

### 3.5 Presence of Rsm binding sites upstream of LuxR transcriptional regulators

**Figure 6:**
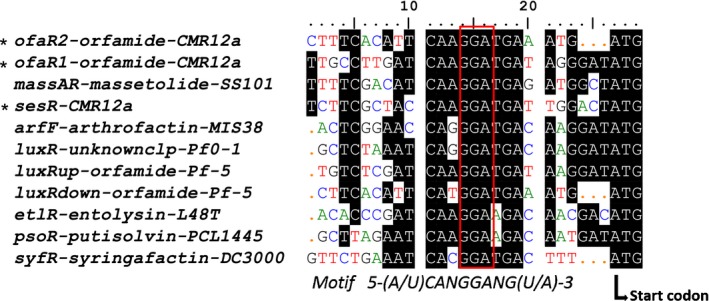
Alignment of the regions upstream of the LuxR transcriptional regulatory genes which flank different lipopeptide biosynthesis gene clusters including *Pseudomonas fluorescens* SS101, *Pseudomonas* sp. MIS38, *P. fluorescens* Pf0‐1, *P. protegens* Pf‐5, *P. putida* PCL1445, *P. entomophilia* L48T, *P. syringae* pv. tomato DC3000, and our study strain *Pseudomonas* sp. CMR12a. The conserved GGA motif is highlighted in red. The translation initiation ATG codon is indicated at the 3′ end, while * indicates the sequences of the test strain used in this study

Genomic search for putative Rsm binding sites was conducted within the sequences upstream of the three *luxR* regulatory genes of CMR12a. Conserved GGA motifs upstream of the ATG start codon could be identified. Sequence alignment of these sequences with their homologs in CLP‐producing *Pseudomonas* strains showed the similarity of these regions upstream of sessilins and orfamide biosynthetic gene clusters with those of previously described CLPs (Figure 6).

## 4 DISCUSSION

Our study revealed that the LuxR‐like transcriptional regulators, OfaR1 and OfaR2, which are associated with the orfamide gene cluster not only regulate orfamide biosynthesis but also sessilin biosynthesis, while we could not find a clear function for the LuxR‐like regulator, SesR, associated with the sessilin gene cluster.

LC‐ESI‐MS analysis revealed that orfamide and sessilin production commences concurrently in the late exponential phase, but orfamide is mainly retained inside the cell and secreted much later and in lower amounts than sessilin. We have previously shown that the release of orfamide in the environment is hampered by sessilin and hypothesized that both compounds compete for the same outer membrane efflux transporter, SesT (D'aes et al., 2014). Here, we show that the *sesT* gene, located upstream of the sessilin biosynthetic cluster, is expressed from an operon together with *sesR*. A mutation in *sesR*, however, does not seem to affect CLP production or secretion. We are currently investigating the secretion of orfamides and sessilins in more detail by mutant analysis of putative transport genes including *macAB*,* sesT*, and *sesD*. In contrast, *ofaR*1 and *ofaR*2 mutants completely lost the capacity to produce both sessilins and orfamides as evidenced by the absence of swarming, lack of a white line‐in‐agar phenotype, and confirmed by LC‐ESI‐MS analysis. Also, in the biocontrol strain *P. fluorescens* SBW25, mutations in the LuxR‐type regulatory genes *viscAR* and *viscBC*R, located up‐ and downstream of the viscosin biosynthesis cluster, led to a loss of viscosin production (De Bruijn & Raaijmakers, 2009a). Other homologs of *ofaR*1, located upstream of their NRPS genes, have been shown to be necessary for the production of putisolvin (*psoR*) (Dubern et al., 2008), arthrofactin (*arfF*) (Washio et al., 2010), and entolysin (*etlR*) (Vallet‐Gely et al., 2010).

So far, coregulation of different classes of CLPs in the same strain has only been demonstrated for plant pathogenic *Pseudomonas* bacteria. In the bean pathogen *P. syringae* pv. *syringae* B728a, three LuxR‐like proteins, SalA, Syrf, and SyrG, were shown to control the biosynthesis of the CLPs syringopeptin and syringomycin (Vaughn & Gross, 2016). SalA controls the expression of both *syrG* and *syrF* (Lu, Scholz‐Schroeder, & Gross, 2002). Furthermore, qRT‐PCR analysis of deletion mutants in *syrF* and *syrG* showed that both genes require a functional *salA* gene for activation. In addition, SyrG appears to function as an upstream transcriptional activator of *syrF* (Vaughn & Gross, 2016). The situation in *Pseudomonas* sp. CMR12a is different since a mutation in either *ofaR1*,* ofaR2*, or *sesR* did not abolish the transcription of the other, although the transcript of *ofaR1* may seem weaker in the *ofaR2* mutant. Our method does not allow precise transcript quantification and further investigation by quantitative RT‐PCR is needed. Likewise in the viscosin producing strain—*P. fluorescens* SBW25, a mutation in either *viscAR* or *viscBCR*,* luxR* genes located up‐ and downstream of the *viscABC* biosynthesis genes, did not substantially affect the transcription of the other (De Bruijn & Raaijmakers, 2009a) indicating that both LuxR regulators do not transcriptionally affect each other.

Transcriptional analyses showed that for both the sessilin and orfamide gene clusters, their biosynthesis genes, *sesABC and ofaABC*, together with putative transport genes, *macAB*, are most likely transcribed from a polycistronic operon, which is probably regulated by OfaR1 and OfaR2. The absence of a *sesC* transcript in CMR12a could be due to primer specificity problems since a coexpression was observed for *sesB‐sesC* and *sesC‐macA1*. With respect to the orfamide gene cluster, worthy of note was the fact that mutants in *ofaR1* and *ofaR2* still showed clear transcripts for *ofaA* and *macA2B2* genes. These results indicate that besides the single promoter which enables the transcription of *ofaABC* and *macA2B2* genes, separate promoters for *ofaA* and *macA2B2* may be present, which are not controlled by OfaR1 and OfaR2. Unfortunately, little information is available about the gene coexpression of other CLP gene clusters except for WLIP (Rokni‐Zadeh et al., 2012), so we could not ascertain if the presence of multiple promoters as was observed in the orfamide gene cluster is a frequent occurrence. In this respect, it is interesting to notice that in beneficial *Pseudomonas* spp., the genomic region encoding the first CLP biosynthesis gene is often unlinked with the other two biosynthesis genes, which are coexpressed. This is for instance the case for viscosin, massetolide, WLIP, xantholysin, entolysin, and poaeamide (De Bruijn et al., 2007, 2008; Li et al., 2013; Rokni‐Zadeh et al., 2012; Vallet‐Gely et al., 2010; Zachow et al., 2015).

During this study, we were unable to complement the CMR12a‐∆*ofaR2* mutant. Considering the fact that the *macB2* gene associated with the orfamide gene cluster gave a weaker transcript than *macA2* for CMR12a, it is possible that *ofaR2* is transcribed from a longer transcript which spans across part of the *macB2* gene. This would result in an antisense overlap that could influence the expression of *macB2* by transcription attenuation (Sesto, Wurtzel, Archambaud, Sorek, & Cossart, 2012). This obviously requires further investigation.

In our study, phylogenetic analysis of LuxR‐type proteins, positioned up‐ and downstream of the CLP gene clusters of CMR12a together with previously described CLP‐associated LuxR regulators revealed that OfaR1 and SesR clustered together with known LuxR‐type regulators located upstream of the CLP biosynthesis genes, whereas OfaR2 clustered with those located downstream. LuxR regulators from strains which produce similar CLPs, for example, orfamide producers *P. protegens* Pf‐5 and *Pseudomonas* sp. CMR12a, cluster together. An exception is the LuxR regulator for poaeamide, *P. poae* RE*1‐1‐14 which although shares a structural relationship with orfamide (Zachow et al., 2015), clusters with LuxR regulators of CLPs belonging to the viscosin family. The LuxR regulator (WipR) of the WLIP producer—*P. reactans* LMG 5329, showed a higher homology with LuxR regulators of the viscosin family compared with that of another WLIP producer—*P. putida* RW10S2 (Rokni‐Zadeh et al., 2013). This decreased conservation suggests that the biosynthetic gene cluster of poaeamide might have evolved separately. Our results further indicate that LuxR‐type regulators of CMR12a belong to the same subfamily as in other plant beneficial *Pseudomonas* strains including *P. protegens* Pf‐5, *P. fluorescens* SS101, and *P. fluorescens* SBW25, which produce orfamide, massetolide, and viscosin, respectively (De Bruijn & Raaijmakers, 2009a; De Bruijn et al., 2008; Loper & Gross, 2007). Given that LuxR transcriptional regulators of *P. syringae* pv. *syringae* cluster with all LuxR regulators analyzed during this study, our results indicate that similar to this plant pathogenic strain, these other LuxR regulators, including OfaR1, OfaR2, and SesR, belong to the fourth LuxR family which is characterized by the absence of any defined N‐terminal domain (Vaughn & Gross, 2016).

During this study, a genomic search and subsequent alignment of sequences upstream of *ofaR1*,* ofaR2*, and *sesR* with their homologs in other lipopeptide biosynthesis genes of *Pseudomonas* strains, showed that Rsm binding sites were located upstream of all three *luxR*‐like genes of CMR12a. Given the fact that this Rsm binding site, alternatively called the GacA box, was found upstream of multiple CLP biosynthesis genes (Song, Voort, et al., 2015a) in different *Pseudomonas* strains, our results suggest that the Gac/Rsm‐mediated regulation of CLPs might be a general phenomenon in most biocontrol CLP‐producing *Pseudomonas* spp.

In conclusion, this study establishes that sessilin and orfamide production in CMR12a are coregulated by two of the three *luxR*‐type genes namely *ofaR1* and *ofaR2*. Our findings show that either OfaR1 or OfaR2 can regulate the biosynthesis of these two CLPs, while the function of SesR remains unclear.

## CONFLICT OF INTEREST

None declared.
