# Supplementary material for: Coregulation of the cyclic lipopeptides orfamide and sessilin in the biocontrol strain Pseudomonas sp. CMR12a
Source: Microbiologyopen. 2017 Jun 15;6(5):e00499. doi: 10.1002/mbo3.499 (PMC5635164; doi:10.1002/mbo3.499)
Supplement: Supplementary file 1 [file MBO3-6-na-s001.docx]

| **Table S1**. Primers used for RT-PCR of the sessilin and orfamide biosynthesis cluster and flanking sequences. | | | | | |
| --- | --- | --- | --- | --- | --- |
| Primer* | Gene (s) | Sequence (5'―3') | |  |  |
|  |  | Forward Primer | Reverse Primer | Length (bp) | Source |
|  | *Sessilin* |  |  |  |  |
| A | SesT | gacactgggaaagaacgctg | caatgcttacctgacctggc | 412 | This study |
| B | SesT-SesR | ctggatcaactgccgcaag | gtcgcacattcatcgtgagt | 504 | This study |
| C | SesR | catccagctgccaattttgc | gctgatcgacaagttcctgc | 451 | This study |
| D | SesD | aaccggaaaccagcaatgtc | cgatcttgatcttgtcggcc | 420 | This study |
| E | SesA | gttccgtcatatctgcgagc | gcggcccaggtaatcaattt | 532 | This study |
| F | SesA-SesB | ctctgggaatacgccgaaat | gaaagctgtcatgccgatca | 543 | This study |
| G | SesB | ggtcgtcagaatgtggtgtt | caggtagtcacaaatgcggc | 447 | This study |
| H | SesB-SesC | aggtggagcacgtggtgg | acgttgaagatggatcgggt | 499 | This study |
| I | SesC | gatttcccaggtgcgtgtac | cgtcagaagcgatatggtgc | 560 | This study |
| J | SesC-MacA1 | cacagggcaatcaacgtgag | cgagcttgactttcagcgat | 538 | This study |
| K | MacA1 | cttgcaggggatcaaacagg | cagtttgccgtagtagcgtt | 613 | This study |
| L | MacA1-MacB1 | caaaccctgatcatcgcctg | atctcgacgttatgcatggc | 609 | This study |
| M | MacB1 | gaccacaacacccgtaatcg | gcctccaccagaaactgttg | 515 | This study |
|  |  |  |  |  |  |
|  | *Orfamide* |  |  |  |  |
| N | OfaR1 | ttgttgccgatactgttggg | gggacagttgatctccagca | 463 | This study |
| O | OfaA | ctggacctgtatcgcacctt | gcttcgccgtagtggtagtc | 499 | This study |
| P | OfaA-OfaB | ccaatacccgcagtcattcg | agcatgtccggagtgatctt | 583 | This study |
| Q | OfaB | gtgacgatgctgttcctcac | gtacggtacaacaacgcacc | 440 | This study |
| R | OfaB-OfaC | gtgaacctgatcagccgga | gcgaactgtgcctgcatc | 540 | This study |
| S | OfaC | gtccaggaccatatcgaggg | ttgttcgagatccaccactg | 585 | This study |
| T | OfaC-MacA2 | cgcccaatgtctaccacttg | gtggccttggtttccttgag | 613 | This study |
| U | MacA2 | ggtttccgggcaattgaagt | gccgaggatggtgaagtaca | 547 | This study |
| V | MacA2-MacB2 | ctgttcgatgtgcccaacc | ggcaaccgaggatgttcatc | 464 | This study |
| W | MacB2 | cattctccaggagctgcatg | atgtcgcggaccttgaagta | 600 | This study |
| X | OfaR2 | ggtagctctcgacggtgtt | ccgtgcacaaccatcatcc | 492 | This study |

*Designation for primer positions as indicated on the biosynthetic clusters in Figures 1A and 1B.

**Table S2**. Characteristics of strains and protein sequences used in the phylogenetic analyses of LuxR proteins of CLP-producing Pseudomonas spp.

| **Strain** | **Associated CLP** | **Protein Annotation** | **Accession Number** | **Reference** |
| --- | --- | --- | --- | --- |
| *P. poae* RE*1-1-14 | poaemide | LuxR | WP_003235573 | Müller *et al.,* 2013 |
| *P. poae* RE*1-1-14 | poaemide | LuxR | WP_011059319.1 | Müller *et al.,* 2013 |
| *P. protegens* Pf-5 | orfamide | PFL_2143-LuxRup | [AAY91417](https://www.ncbi.nlm.nih.gov/protein/68343811) | Paulsen *et al.,* 2005 |
| *P. protegens* Pf-5 | orfamide | PFL_2150-LuxRdown | [AAY91424](https://www.ncbi.nlm.nih.gov/protein/68343818) | Paulsen *et al.,* 2005 |
| *P. fluorescens* Pf0-1 | undescribed | PFL01_RS11145 | [WP_011333638](https://www.ncbi.nlm.nih.gov/protein/499652904) | Silby *et al.,* 2009 |
| *P. fluorescens* Pf0-1 | undescribed | PFL01_RS11175 | [WP_041475215](https://www.ncbi.nlm.nih.gov/protein/752846204) | Silby *et al.,* 2009 |
| *Pseudomonas sp.* MIS38 | arthrofactin | ArfF | BAC67533 | Washio *et al.,* 2010 |
| *P. putida* BW11M1 | xantholysin | XtlR | [AGM14924](https://www.ncbi.nlm.nih.gov/protein/506444276) | Li *et al.,* 2013 |
| *P. entomophila* L48T | entolysin | EtlR | [WP_011534474](https://www.ncbi.nlm.nih.gov/protein/499853740) | Vodovar *et al.,* 2006 |
| *P. putida* PCL1445 | putisolvin | PsoR | [ABW17374](https://www.ncbi.nlm.nih.gov/protein/158137910) | Dubern *et al.,* 2006 |
| *P. putida* PCL1445 | putisolvin | LuxR | ABW17380 | Dubern *et al.,* 2006 |
| *P. putida* RW10S2 | WLIP | WlpR | AFJ23818 | Rokni-Zadeh *et al.,* 2012 |
| *P*. *syringae* pv. *syringae* DC3000 | syringopeptin | SyrR | [NP_792632](https://www.ncbi.nlm.nih.gov/protein/28870013) | Buell *et al.,* 2003 |
| *P*. *syringae* pv. *syringae* DC3000 | syringostatin | LuxR | [NP_792637](https://www.ncbi.nlm.nih.gov/protein/28870018) | Buell *et al.,* 2003 |
| *P. fluorescens* SBW25 | viscosin | ViscAR | WP_015884801 | Silby *et al.,* 2009 |
| *P. fluorescens* SBW25 | viscosin | PFLU_2557-ViscBCR | [WP_043205227](https://www.ncbi.nlm.nih.gov/protein/759484298) | Silby *et al.,* 2009 |
| *P. fluorescens* SS101 | massetolide | MassAR | WP_003192380 | Loper *et al.,* 2012 |
| *P. fluorescens* SS101 | massetolide | MassBCR | WP_032900559 | Loper *et al.,* 2012 |
| *Pseudomonas* sp. CMR12a | orfamide | OfaR1 | AFH75327 | D’aes *et al.,* 2014 |
| *Pseudomonas* sp. CMR12a | orfamide | OfaR2 | AFH75333 | D’aes *et al.,* 2014 |
| *Pseudomonas* sp. CMR12a | sessilin | SesR | AFH75318 | D’aes *et al.,* 2014 |
| *P. tolaasii* PMS117 | tolaasin | LuxR | WP_016969117 | Studholme *et al.,* unpublished |
| *P. constantinii* DSM 16734 | tolaasin | LuxR | CCJ67634 | Graupner *et al.,* unpublished |
| *P. fluorescens* In5 | nunamycin | NunF | AHL29304 | Michelsen *et al.,* 2015 |
| *P. syringae* pv. *syringae* B301D | syringomycin and syringopeptin | SyrF | WP_011267816 | Ravindran *et al.,* 2015 |
| *P. syringae* pv. *syringae* B301D | syringomycin and syringopeptin | SyrG | WP_032656102 | Ravindran *et al.,* 2015 |
| *P. syringae* pv. *syringae* B301D | syringomycin and syringopeptin | SalA | WP_024638707 | Ravindran *et al.,* 2015 |
| *Pseudomonas* sp. CMR12a | - | CmR | ADN34749 | De Maeyer *et al.,* 2011 |
| *Pseudomonas* sp. CMR12a | - | PhzR | ADN06870 | De Maeyer *et al.,* 2011 |
| *Vibrio fischeri* | - | LuxR | AAQ90196 | Nishiguchi and Nair *et al.,* 2003 |

**References**

Buell, C. R., Joardar, V., Lindeberg, M., Selengut, J., Paulsen, I. T., Gwinn, M. L., Dodson, R. J., Deboy, R. T., Durkin, A. S., Kolonay, J. F., Madupu, R., Daugherty, S., Brinkac, L., Beanan, M. J., Haft, D. H., Nelson, W. C., Davidsen, T., Zafar, N., Zhou, L., Liu, J., Yuan, Q., Khouri, H., Fedorova, N., Tran, B., Russell, D., Berry, K., Utterback, T., Van Aken, S. E., Feldblyum, T. V., D'Ascenzo, M., Deng, W., Ramos, A. R., Alfano, J. R., Cartinhour, S., Chatterjee, A. K., Delaney, T. P., Lazarowitz, S. G., Martin, G. B., Schneider, D. J., Tang, X., Bender, C. L., White, O., Fraser, C. M., and Collmer, A. (2003) The complete genome sequence of the arabidopsis and tomato pathogen *Pseudomonas syringae* pv. tomato DC3000. *Proceedings of the National Academy of Sciences* **100**: 10181-10186.

Loper, J. E., Hassan, K. A., Mavrodi, D. V., Davis, E. W., Lim, C. K., Shaffer, B. T., Elbourne, L. D. H., Stockwell, V. O., Hartney, S. L., Breakwell, K., Henkels, M. D., Tetu, S. G., Rangel, L. I., Kidarsa, T. A., Wilson, N. L., van de Mortel, J. E., Song, C., Blumhagen, R., Radune, D., Hostetler, J. B., Brinkac, L. M., Durkin, A. S., Kluepfel, D. A., Wechter, W. P., Anderson, A. J., Kim, Y. C., Pierson III, L. S., Pierson, E. A., Lindow, S. E., Kobayashi, D. Y., Raaijmakers, J. M., Weller, D. M;, Thomashow, L. S., Allen, A. E., and Paulsen, I. T. (2012) Comparative genomics of plant-associated *Pseudomonas* spp.: Insights into diversity and inheritance of traits involved in multitrophic interactions. *PLoS Genetics* **8**: e1002784.

Müller, H., Zachow, C., Alavi, M., Tilcher, R., Krempl, P. M., Thallinger, G. G., and Berg, G. (2013) Complete genome sequence of the sugar beet endophyte *Pseudomonas poae* RE* 1-1-14, a disease-suppressive bacterium. *Genome Announcements* **1**: e00020- 13.10.1128/genomeA.00020-13.

Nishiguchi, M. K. and Nair, V. S. (2003) Evolution of symbiosis in the Vibrionaceae: a combined approach using molecules and physiology. *International Journal of Systematic and Evolutionary Microbiology* **53**:2019–2026.

Paulsen, I. T., Press, C. M., Ravel, J., Kobayashi, D. Y., Myers, G. S. A., Mavrodi, D. V., DeBoy, R. T., Seshadri, R., Ren, Q., Madupu, R., Dodson, R. J., Durkin, A. S., Brinkac, L. M., Daugherty, S. C., Sullivan, S. A., Rosovitz, M. J., Gwinn, M. L., Zhou, L., Schneider, D. J., Cartinhour, S. W., Nelson, W. C., Weidman, J., Watkins, K., Tran, K., Khouri, H., Pierson, E. A., Pierson III, L. S., Thomashow, L. S., and Loper, J. E. (2005) Complete genome sequence of the plant commensal *Pseudomonas fluorescens* Pf-5. *Nature Biotechnology* **23**(7): 873-878.

Ravindran, A., Jalan, N., Yuan, J. S., Wang, N. and Gross, D. C. (2015) Comparative genomics of *Pseudomonas syringae* pv. *syringae* strains B301D and HS191 and insights into intrapathovar traits associated with plant pathogenesis. *MicrobiologyOpen* **4**(4): 553-573.

Silby, M. W., Cerdeno-Tarraga, A. M., Vernikos, G. S., Giddens, S. R., Jackson, R. W., Preston, G. M., Zhang, X. -X., Moon, C. D., Gehrig, S. M., Godfrey, S. A. C., Knight, C. G., Malone, J. G., Robinson, Z., Spiers, A. J., Harris, S., Challis, G. L., Yaxley, A. M., Harris, D., Seeger, K., Murphy, L., Rutter, S., Squares, R., Quail, M. A., Saunders, E., Mavromatis, K., Brettin, T. S., Bentley, S. D., Hothersall, J., Stephens, E., Thomas, C. M., Parkhill, J., Levy, S. B., Rainey, P. B., and Thomson, N. R. (2009) Genomic and genetic analyses of diversity and plant interactions of *Pseudomonas fluorescens*. *Genome Biology* **10**(5): R51.

Vodovar, N., Vallenet, D., Cruveiller, S., Rouy, Z., Barbe, V., Acosta, C., Cattolico, L., Jubin, C., Lajus, A., Segurens, B., Vacherie, B., Wincker, P., Weissenbach, J., Lemaitre, B., Médigue, C., and Boccard, F. (2006) Complete genome sequence of the entomopathogenic and metabolically versatile soil bacterium *Pseudomonas entomophila*. *Nature Biotechnology* **24**: 673–679.
